# Differential Role of TGF-β in Extracellular Matrix Regulation During *Trypanosoma cruzi*-Host Cell Interaction

**DOI:** 10.3390/ijms20194836

**Published:** 2019-09-29

**Authors:** Tatiana Araújo Silva, Luis Felipe de Carvalho Ferreira, Mirian Claudia de Souza Pereira, Claudia Magalhães Calvet

**Affiliations:** Cellular Ultrastructure Laboratory, Oswaldo Cruz Institute, FIOCRUZ, Rio de Janeiro 21040-360, Brazil; tatibio86@gmail.com (T.A.S.); lfdcferreira@gmail.com (L.F.d.C.F.); mirian@ioc.fiocruz.br (M.C.d.S.P.)

**Keywords:** *Trypanosoma cruzi*, TGF-β, heart fibrosis, extracellular matrix, signaling pathways, SMAD2, p38 MAPK, c-Jun

## Abstract

Transforming growth factor beta (TGF-β) is a determinant for inflammation and fibrosis in cardiac and skeletal muscle in Chagas disease. To determine its regulatory mechanisms, we investigated the response of *Trypanosoma cruzi*-infected cardiomyocytes (CM), cardiac fibroblasts (CF), and L6E9 skeletal myoblasts to TGF-β. Cultures of CM, CF, and L6E9 were infected with *T. cruzi* (Y strain) and treated with TGF-β (1–10 ng/mL, 1 h or 48 h). Fibronectin (FN) distribution was analyzed by immunofluorescence and Western blot (WB). Phosphorylated SMAD2 (PS2), phospho-p38 (p-p38), and phospho-c-Jun (p-c-Jun) signaling were evaluated by WB. CF and L6E9 showed an increase in FN from 1 ng/mL of TGF-β, while CM displayed FN modulation only after 10 ng/mL treatment. CF and L6E9 showed higher PS2 levels than CM, while p38 was less stimulated in CF than CM and L6E9. *T. cruzi* infection resulted in localized FN disorganization in CF and L6E9. *T. cruzi* induced an increase in FN in CF cultures, mainly in uninfected cells. Infected CF cultures treated with TGF-β showed a reduction in PS2 and an increase in p-p38 and p-c-Jun levels. Our data suggest that p38 and c-Jun pathways may be participating in the fibrosis regulatory process mediated by TGF-β after *T. cruzi* infection.

## 1. Introduction

Chagas disease (CD), caused by the flagellated protozoan *Trypanosoma cruzi* [1], currently affects 6–8 million people worldwide [2]. The disease scenario has shifted to a global threat due to migration of infected individuals to non-endemic areas [3,4]. CD presents in acute and chronic clinical forms. While the former is typically inapparent or presents only mild manifestations and is commonly ignored due to non-specific signs, the latter, although asymptomatic in approximately 70% of cases, may progress to severe damage. Muscular pain and weakness are also frequent symptoms in Chagasic patients [5]. Chronic Chagas cardiomyopathy (CCC), estimated to be present in 20–30% of infected individuals [6,7], is the most severe manifestation in CD with a high morbidity and mortality and has significant economic and social impacts in Latin America [8].

In CD, cardiomyopathy is characterized by an intense myocarditis related to the presence of inflammatory cells reacting to the parasite, resulting in cardiac tissue damage and fibrosis [9]. Skeletal muscle tissue also presents parasitism and inflammation in myofibers [5,10]. Cardiac fibroblasts are the main actors in fibrotic processes acting on the remodeling of the extracellular matrix (ECM) in the myocardium [11]. External stimuli, including chemokines and cytokines, regulate fibroblast proliferation and ECM synthesis and secretion [12].

Transforming growth factor β (TGF-β), a cytokine involved in various biological and physiological processes [13], has been highlighted as a regulator of the fibrotic process in the pathogenesis of Chagas disease [14,15]. The signaling pathway of the TGF-β family occurs through three receptors, type I (TGFβI), II (TGFβII), and III (TGFβIII). Type III receptors, also called betaglycans or endoglycans, act as co-receptors, presenting the cytokine to its specific receptors. The binding of active TGF-β to the TGFβII propagates the signal via downstream effectors with the phosphorylation of proteins from the TGF-β classical (canonical) signaling pathway and SMAD proteins (SMADS 1–7) [16]. In addition to the classical signaling pathway, TGF-β also modulates non-canonical signaling pathways, such as p38 mitogen-activated protein kinase (p38 MAPK), extracellular signal regulated kinase (ERK), c-Jun N-terminal kinase (JNK), nuclear factor-kB (NF-kB), and small GTPases [17,18]. JNK and p38 MAPK are the alternative routes of TGF-β signaling best characterized. The JNK and p38 MAPK pathways are activated by MAP kinases (MKKs) MKK4 and MKK 3/6, respectively. TGF-β also triggers the activation of TGF-β1-associated kinases (TAK1) through the catalytic activation of factor-associated tumor necrosis factor receptor 6 (TRAF6), crucial processes for the activation of JNK and p38 MAPK [19].

TGF-β plays an important role in *T. cruzi* biology. This cytokine is involved in the host cell invasion process, since *T. cruzi* requires functional TGF-β receptors and activation of its classical signaling pathway to invade the host cell [20,21,22]. Also, amastigote forms capture TGF-β from the host cell, using it for differentiation into the trypomastigote form, which allows the completion of the parasite intracellular cycle [23]. Experimental evidence also shows that the parasite induces the synthesis of TGF-β in cardiomyocytes and cardiac fibroblasts [24], which may influence the survival of the parasite. Trypomastigote and amastigote forms are able to directly activate latent TGF-β through its main cysteine peptidase (CP), cruzipain, [25,26], which may contribute to the invasion process and the genesis of Chagas disease.

In addition, the host immune response is controlled by TGF-β during *T. cruzi* infection [27,28,29]. Elevated TGF-β levels are associated with intense myocardial fibrosis detected in *T. cruzi*-infected α2-macrobulin knockout mice [28] and in patients with CCC [14]. TGF-β receptor expression is increased in *T. cruzi*-infected mouse hearts in association with increased collagen I and fibronectin expression in myocardium [30]. Recent reports describe that TGF-β inhibitors reduce infection and prevent cardiac damage and fibrosis during experimental *T. cruzi* infection [31,32,33,34,35]. Taken together, these evidences show a key role of TGF-β in *T. cruzi* infection and in Chagas disease cardiomyopathy.

Besides the known enhancement of ECM during fibrosis process in vivo, our group demonstrated that cardiomyocytes highly infected with *T. cruzi* (72 and 96 h of infection) in vitro present low fibronectin expression, while adjacent non-infected cells show an intense network of extracellular matrix component similar to the control [36], even after treatment with TGF-β [33]. Although TGF-β treatment is able to trigger the increase of ECM expression in uninfected cardiomyocytes, the effect is dose dependent, being observed only after the treatment with high concentrations of TGF-β (>10 ng/mL) [33].

Given that cardiac fibroblasts, the main cell type responsible for ECM synthesis in the myocardium, are the effector cells in the cardiac fibrosis process, [37], and that cell lines originated from skeletal muscle (skeletal myoblasts L6E9) respond to TGF-β in picomolar concentrations [38], we evaluated the expression of extracellular matrix components in uninfected and *T. cruzi*-infected cardiac fibroblasts and skeletal muscle cells (L6E9) after treatment with different concentrations of TGF-β and analyzed the underlying signaling mechanisms of these processes.

## 2. Results

### 2.1. Modulation of Fibronectin Spatial Distribution in Different Cell Types after TGF-β Stimulation

**Figure 1:**
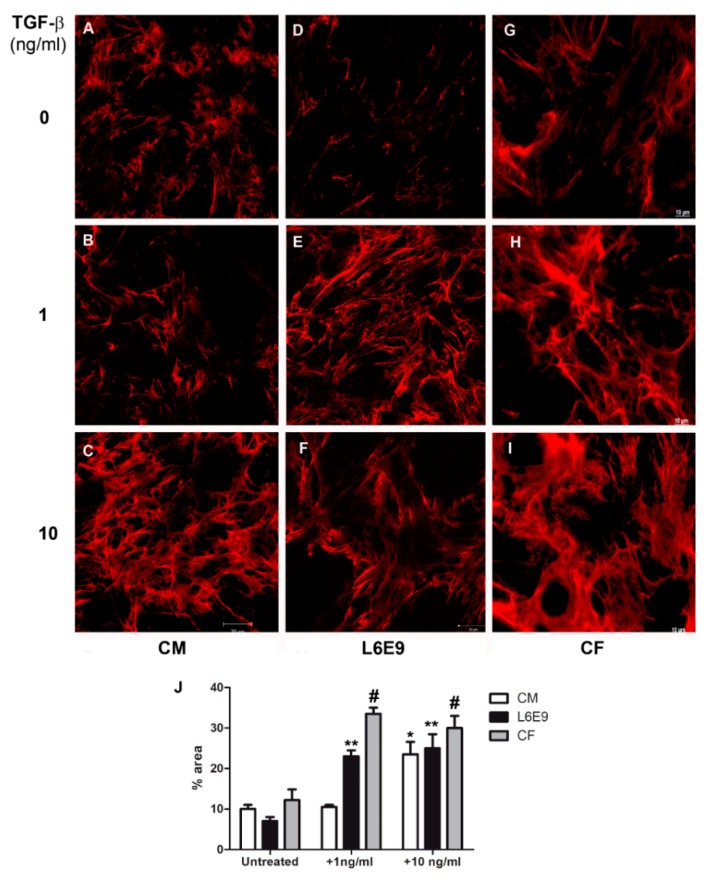
Transforming growth factor beta (TGF-β) differentially modulates fibronectin (FN) in cardiomyocytes, skeletal myoblasts L6E9, and cardiac fibroblasts. (**A**) Normal cardiomyocytes showed FN fibrils on surface. The addition of 1 ng/mL of TGF-β (**B**) did not alter the FN expression in normal cardiomyocytes. Only 10 ng/mL of TGF-β induced the increase of FN expression (**C**). In contrast, L6E9 skeletal myoblasts (**E**) and cardiac fibroblasts (**H**) presented the FN matrix stimulation after treatment with 1 ng/mL TGF-β when compared to untreated cultures (**D**,**G**). The FN increase was still detected in these two cell types with addition of 10 ng/mL (**F**,**I**) of TGF-β. (**J**) FN staining area was measured with Image J software, showing the thickening of FN fibrils after TGF-β treatment. * *p* ≤ 0.05 compared to untreated cardiomyocytes (CM); ** *p* ≤ 0.05 compared to untreated L6E9; # *p* ≤ 0.05 compared to untreated cardiac fibroblasts (CF). Bar = 20 μm.

In an attempt to understand the mechanisms underlying cardiac fibrosis evidenced in the pathogenesis of Chagas disease, we evaluated the modulation of extracellular matrix components in cardiomyocytes (CM), cardiac fibroblasts (CFs), and L6E9 skeletal myoblasts stimulated with TGF-β, an important mediator of the fibrosis process. First, the distribution of fibronectin (FN) fibrils in the ECM of these cell types was analyzed in response to stimulation of TGF-β (1–10 ng/mL) for 48 h by indirect immunofluorescence. Purified CF cultures were achieved after the fifth culture passage of CM cultures, as determined by the absence of Western blot (WB) reactivity for desmin, intermediate filament proteins specifically expressed in CM (Supplemental Material, Figure S1). Confocal microscopy revealed the arrangement of FN fibrils on the CM surface, evidencing a thickening at the FN network dependent on cytokine stimulus concentration (Figure 1). The profile of FN distribution in the ECM remained similar to the untreated cells (Figure 1A) even after stimulation with TGF-β at 1 ng/mL (Figure 1B). However, CMs stimulated with 10 ng/mL of TGF-β (Figure 1C) displayed an increase in the thickness of FN fibrils in the ECM, as previously reported [33]. Measurement of FN staining area with Image J software (Figure 1J) showed an increase from an average of 10% of total area (untreated CM, Figure 1A) and 10.5% (CM + 1 ng/mL TGF-β, Figure 1B) to 24% of total area after treatment with 10 ng/mL TGF-β, a 2.3-fold increase in FN fibril thickness. In contrast, an increase in FN fibrils was revealed at the surface of L6E9 skeletal myoblasts (Figure 1E) and CFs (Figure 1H) after stimulation with 1 ng/mL of TGF-β, a dose 10 times lower than that required to stimulate CMs. The enhancement of FN deposit occurred in a dose-dependent manner, showing thicker FN fibrillar network after treatment of L6E9 skeletal myoblasts (Figure 1F) and CF (Figure 1I) with 10 ng/mL of the cytokine. Quantification of FN fibril thickness by image processing with Image J software (Figure 1J) showed an increase of 3.2-fold of FN staining area after addition of 1 ng/mL TGF-β and 3.5-fold after 10 ng/mL for L6E9 skeletal myoblasts. In a similar fashion, CFs displayed 2.8-fold increase of FN fibrils after treatment with 1 ng/mL of TGF-β and 2.3-fold increase after addition of 10 ng/mL of the cytokine (Figure 1J).

### 2.2. Signaling Pathways Triggered by TGF-β Stimulation

**Figure 2:**
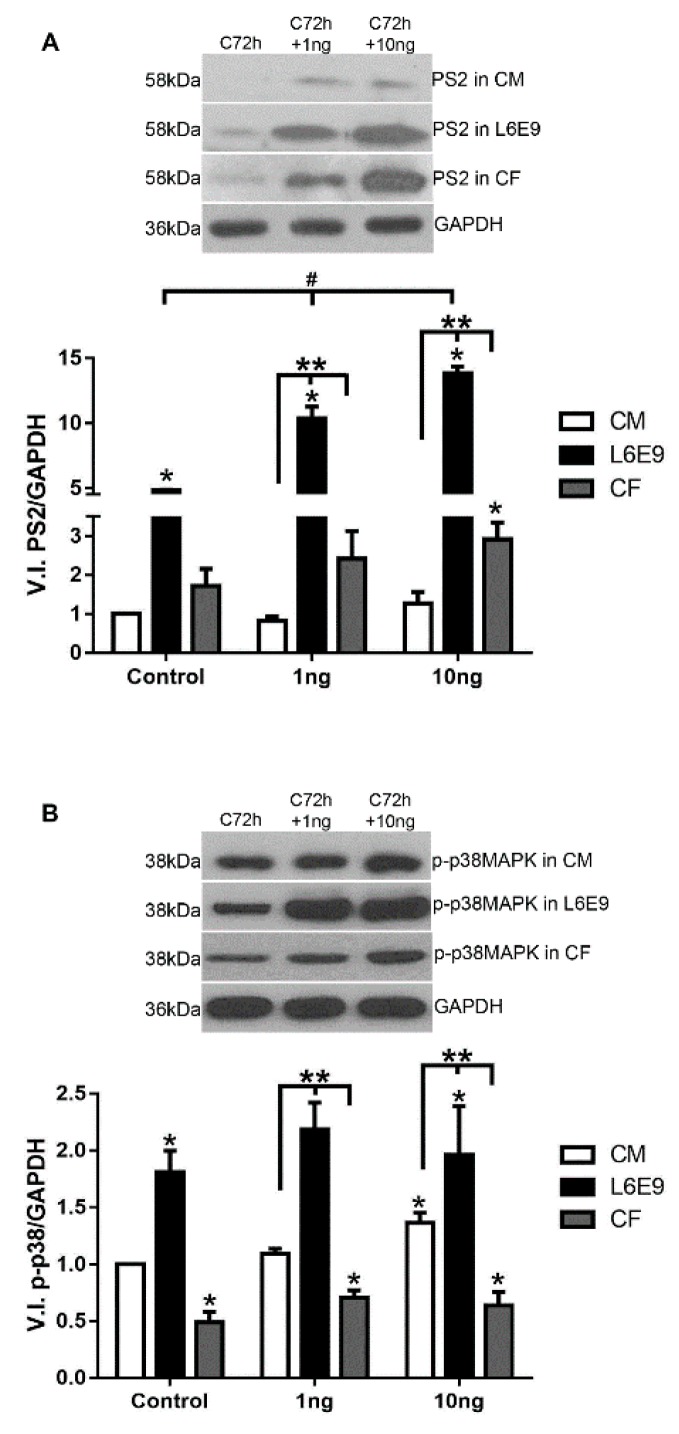
Phosphorylated SMAD2 and p38 MAPK detection in normal cultures. (**A**) Comparison of SMAD2 phosphorylation (PS2) detection of CM, L6E9 skeletal myoblasts, and CF treated with TGF-β (1–10 ng/mL) per 1 h. PS2 basal levels in L6E9 skeletal myoblasts and CF were significantly greater than CM. After the addition of TGF-β, PS2 remained significantly higher in L6E9 and CF relative to CM. L6E9 skeletal myoblasts responded to TGF-β stimulus in a dose dependent manner. CF showed a slight level of PS2 increase. * *p* ≤ 0.05 compared to CM; ** *p* ≤ 0.05 compared with CM in a different concentration; # *p* ≤ 0.05 compared with L6E9 control. *N* = 3; (**B**) Comparison of phosho-p38 MAPK detection of CM, L6E9 skeletal myoblasts, and CF treated with TGF-β (1–10ng/mL) per 1 h. L6E9 skeletal myoblasts presented a high level of p38 MAPK phosphorylation when compared with CM. When CM was compared with CF, a higher p38 MAPK phosphorylation after stimulation with TGF-β (1–10 ng/mL) was observed. * *p* ≤ 0.05 compared to CM and ** *p* ≤ 0.05 compared with CM in a different concentration. *N* = 3.

The discrepancy in response to TGF-β stimulation led us to investigate whether the distinct modulation of FN profile between CFs, L6E9 skeletal myoblasts, and CMs could be related to different signaling pathways triggered in the ECM regulation process. Thus, the classic and the alternative TGF-β signaling pathways were evaluated in all cell types after cytokine treatment, using CM cultures as reference. Comparing the baseline response without TGF-β stimulus, L6E9 SMAD2 phosphorylation (PS2) was 5-fold higher than CM, while CF displayed 1.8-fold the PS2 levels of CM (Figure 2A). Analysis of the SMAD pathway revealed that, following stimulation of L6E9 skeletal myoblasts with 1 and 10 ng/mL of TGF-β, respectively, 2.1- and 2.9-fold increases in levels of PS2 in L6E9 skeletal myoblasts were observed compared to untreated L6E9 cultures (Figure 2A). The kinetics of TGF-β treatment of CFs revealed a 40% increase in PS2 levels after stimulation with 1 ng/mL TGF-β, while with 10 ng/mL stimulation, a maximum level of 69% increase was attained (Figure 2A). Interestingly, L6E9 skeletal myoblasts were more susceptible to cytokine stimulation, presenting levels of PS2 10- to 13-fold higher than CM and 4-fold higher than CFs at the TGF-β concentrations analyzed. CF also presented more PS2 activation than CM, achieving increases of 2.5 and 2.7-fold above CM cultures when stimulated with 1 ng/mL and 10 ng/mL of TGF-β, respectively (Figure 2A). CMs exhibited increases in PS2 level only after high doses of TGF-β (10 ng/mL) (Figure 2A).

In parallel, we also evaluated the non-canonical p38 MAPK phosphorylation (phospho-p38 MAPK, phosho-p38) pathway in CFs, CMs, and L6E9 skeletal myoblasts after 1 h treatment with TGF-β (1–10 ng/mL). L6E9 skeletal myoblasts also showed P-p38 levels 80% higher than CM and 3.7-fold higher than CF at the baseline. L6E9 also showed increased sensitivity to TGF-β compared to CMs and CFs, reaching 2- and 4.7-fold increases in CM and CF levels, respectively, after TGF-β treatment (Figure 2B). An increase in phospho-p38 MAPK levels was observed in CF cultures stimulated with TGF-β, reaching a maximum of 45% increase in concentration at 10 ng/mL (Figure 2B). In contrast, a 37% p38 MAPK activation was noticed in CMs stimulated only with 10 ng/mL of TGF-β. In all concentrations of TGF-β (1–10 ng/mL), the phospho-p38 MAPK levels in CFs were significantly lower than in CMs and L6E9 (Figure 2B). The addition of 1 ng/mL of TGF-β to L6E9 skeletal myoblasts resulted in a 1.2-fold increase of phospho-p38 MAPK, which remained constant after treatment with higher doses of TGF-β (Figure 2B). In contrast, even though the responses of L6E9 and CMs to the cytokine stimulus were similar, with a rise in the range of 20–35%, TGF-β-treated CMs did not reach levels of phospho-p38 MAPK comparable to the profile of stimulated L6E9 skeletal myoblasts, even after high doses of cytokine treatment.

### 2.3. Differential Fibronectin Expression Induced by T. cruzi Infection and TGF-β Stimulation

**Figure 3:**
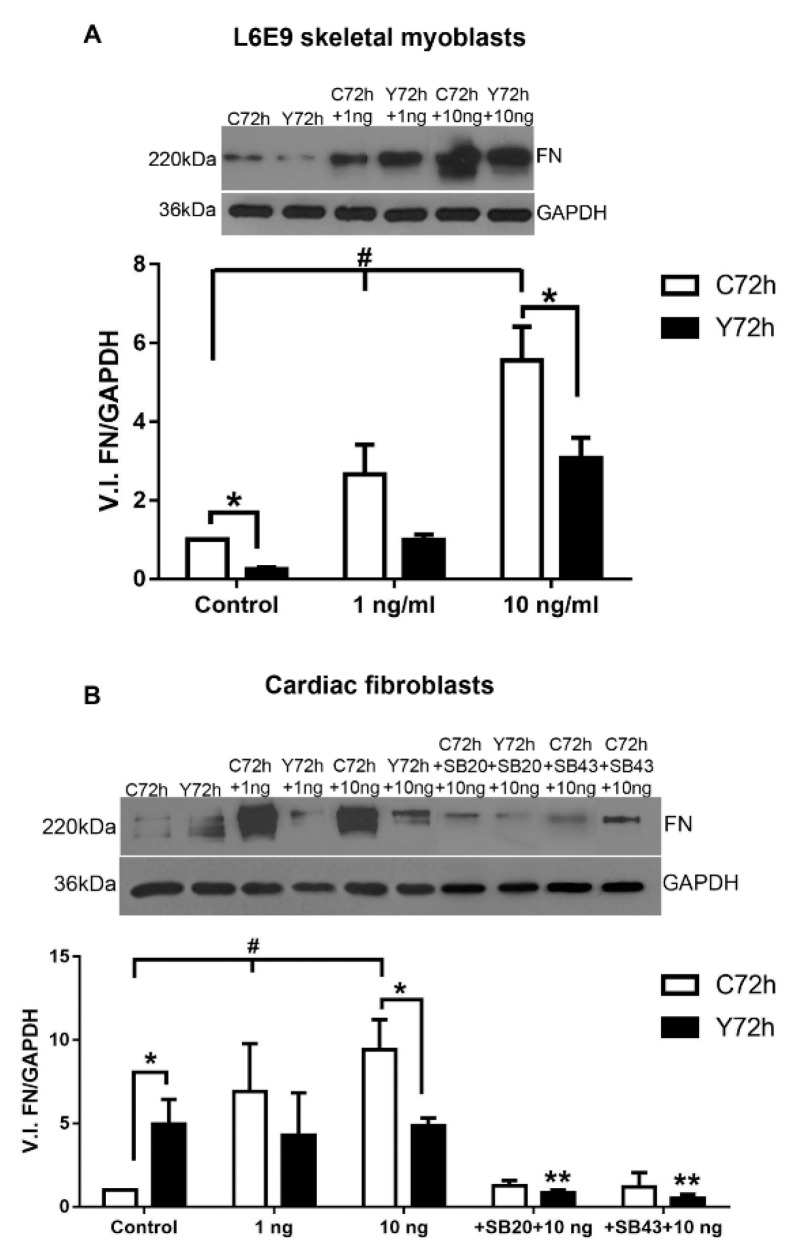
Fibronectin expression in L6E9 skeletal myoblasts and cardiac fibroblasts treated with TGF-β. (**A**) In normal cultures, treatment with 1 ng/mL TGF-β for 48 h triggered the increase of FN expression in L6E9 skeletal myoblasts in a dose-dependent manner after the addition of 1 and 10 ng/mL TGF-β. In L6E9 skeletal myoblasts infected with *T. cruzi* (72 h) and treated with TGF-β (1 and 10 ng/mL) an increase of FN expression was observed when compared with the infected and untreated cultures, but there was a reduction in the FN expression when compared with the uninfected cultures, even after treatment with TGF-β (1 and 10 ng/mL). * *p* ≤ 0.05 compared to uninfected pairs; # *p* ≤ 0.05 compared to untreated control and uninfected culture; *N* = 4. (**B**) Normal CF was treated with TGF-β (1 and 10 ng/mL) and showed an increase of FN expression according to the dose administered. In CF infected and untreated, there was an increase in the FN expression when compared with their normal counterparts. In CF infected and treated with TGF-β (1 and 10 ng/mL), a reduction of FN expression was observed when compared with their treated pairs. Pre-treatment of the culture with p38 (SB203580) and SMAD2 (SB431543) inhibitors prevented FN stimulation by TGF-β. White bars correspond to control, uninfected cultures (C72h), while black bars depict levels of *T. cruzi* (Y strain) infected cultures (Y72h). * *p* ≤ 0.05 compared to uninfected pairs; # *p* ≤ 0.05 compared with control uninfected and untreated and ** *p* ≤ 0.05 compared to TGF-β treated controls (10 ng/mL). *N* = 3.

With the knowledge of the differential modulation in FN expression and the distinct activation of TGF-β–dependent signaling pathways (PS2 and p-p38) in the different cell types, we next sought to evaluate the response of these cells to *T. cruzi* infection. Thus, we evaluated the levels of FN expression in CFs and L6E9 skeletal myoblasts late in *T. cruzi* infection (72 h). Initially, the FN expression of CFs and L6E9 skeletal myoblasts cultures were analyzed after 48 h treatment with TGF-β (1–10 ng/mL) by Western blot. Our results showed increased FN expression in both cell cultures at all TGF-β concentrations analyzed, but CFs were more responsive to cytokine stimulation (Figure 3). L6E9 skeletal myoblasts showed a dose-dependent response with significant increases in FN expression ranging from 3- to 5.5-fold at different concentrations of TGF-β (1 to 10 ng/mL) (Figure 3A). The highest FN increase was observed in CFs, with 6.9-fold increase at 1 ng/mL and 9.4-fold increase at 10 ng/mL, while L6E9 skeletal myoblasts reached a maximum of 5.5-fold enhancement at 10 ng/mL (Figure 3).

Next, we analyzed the expression of FN in L6E9 and CFs infected by *T. cruzi* (72 h). Interestingly, *T. cruzi* infection induced a differential response on FN expression in L6E9 skeletal myoblast and CF cultures (Figure 3). Infected L6E9 skeletal myoblasts showed a significant 82% reduction in FN expression, while CFs demonstrated a 5-fold up-regulation in the expression of this ECM component (Figure 3). Although cytokine treatment stimulated a significant increase in FN expression in *T. cruzi*-infected L6E9 skeletal myoblasts, as compared to non-stimulated infected cells, FN expression levels were significantly down-regulated in comparison to stimulated control cells (Figure 3A). Significant reductions of 67% and 45% in FN expression were demonstrated in *T. cruzi*-infected L6E9 stimulated with 1 and 10 ng/mL TGF-β, respectively, compared to uninfected cultures. In contrast, *T. cruzi*-infected and stimulated CF cultures were not responsive to TGF-β stimulation, even at high concentrations of the cytokine (10 ng/mL), maintaining FN levels similar to control (Figure 3B). In CF cultures with a high degree of infection, even treated with TGF-β, reductions of 38% (1 ng/mL) and 48% (10 ng/mL) in FN expression were observed when compared to their normal treated pairs (Figure 3B). Pre-treatment of CF cultures with signaling inhibitors for 1 h (p38- SB203580, 10 μM; ALK5- SB431542, 10 μM) before the addition of TGF-β prevented the FN stimulation, resulting in FN levels similar to untreated and uninfected controls (Figure 3B). In addition, the treatment of control and *T. cruzi* infected cultures with ALK 5 inhibitor (SB431542) prevented the FN stimulation induced by *T. cruzi*, suggesting that TGF-β triggered by parasite infection modulates FN expression (Supplemental Material, Figure S2).

### 2.4. Regulation of Fibronectin Fibrillar Network Assembly by T. cruzi Infection

**Figure 4:**
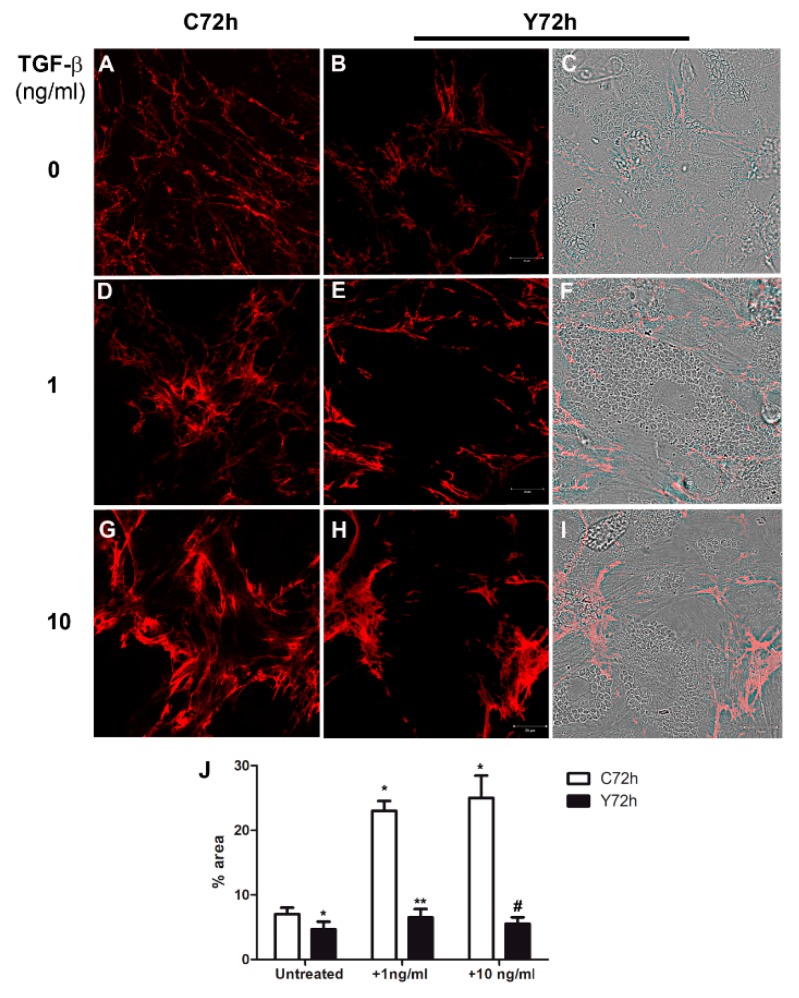
Distribution of fibronectin in L6E9 skeletal myoblasts infected with *T. cruzi* and treated with TGF-β. *T. cruzi* infection (72 h) reduced FN fibrils staining (red) in L6E9 skeletal myoblasts (**B**,**C**) when compared to uninfected controls (**A**). The treatment of *T. cruzi* infected L6E9 skeletal myoblasts with 1 ng/mL (**E**,**F**) or 10 ng/mL (**H**,**I**) of TGF-β induced an increase in FN expression only in uninfected areas in this culture when compared to uninfected TGF-β treated cultures ((**D**) 1 ng/mL; (**G**) 10 ng/mL), which presented widespread enhancement of FN. (**J**) Measurement of FN staining area with Image J software showed a reduction of the FN area after infection and a thickening of FN fibrils after TGF-β treatment in uninfected cultures. Differential interference contrast (DIC) was used to show the intracellular parasites in the host cells. * *p* ≤ 0.05 vs. untreated, uninfected control (C72h); ** *p* ≤ 0.05 vs. uninfected + 1 ng/mL TGF-β; # *p* ≤ 0.05 vs. uninfected + 10 ng/mL TGF-β. Bar = 20 μm.

**Figure 5:**
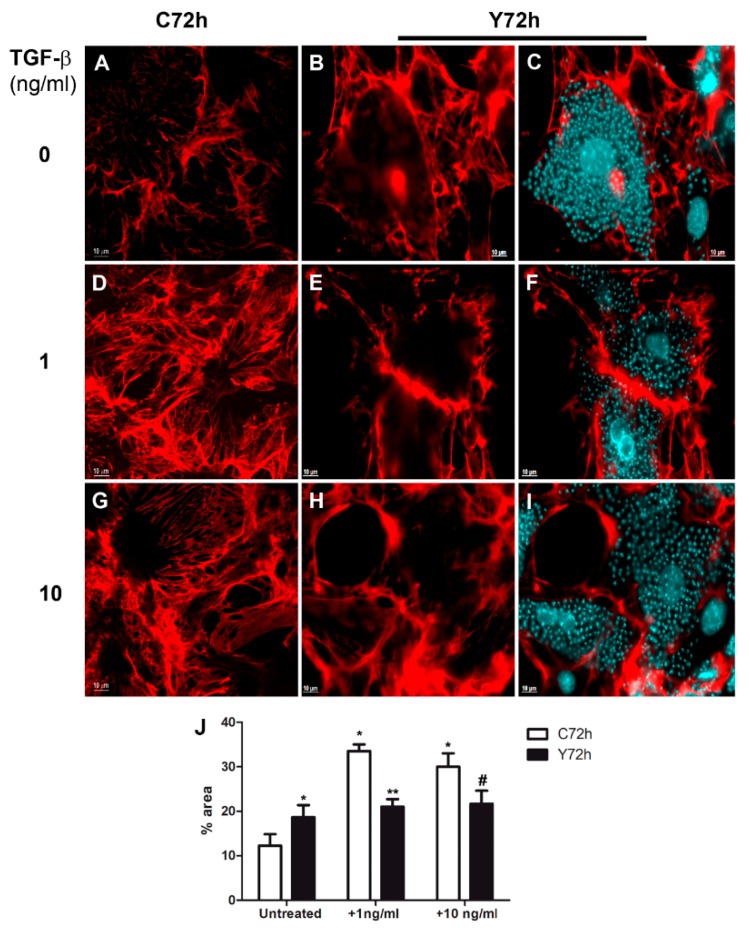
Distribution of fibronectin in cardiac fibroblasts infected with *T. cruzi* and treated with TGF-β. FN staining (red) in untreated *T. cruzi* infected CF (**B**,**C**) showed a general intensification of FN expression after 72 h of interaction, while highly infected cells presented a localized reduction when compared to uninfected controls (**A**). CF cultures showed higher FN expression after treatment with 1 ng/mL (**D**) and 10 ng/mL (**G**) of TGF-β. CF still displayed a localized reduction of FN staining in infected cells, even after TGF-β treatment ((**E**,**F**) 1 ng/mL; (**H**,**I**) 10 ng/mL). (**J**) Quantification of FN fibril thickness by image processing with Image J software. DAPI (blue) was used to label the nucleus of the host cell and the kinetoplast of intracellular parasites. * *p* ≤ 0.05 vs. untreated, uninfected control (C72h); ** *p* ≤ 0.05 vs. uninfected + 1 ng/mL TGF-β; # *p* ≤ 0.05 vs. uninfected + 10 ng/mL TGF-β. Bar = 20 μm.

The FN distribution in the extracellular matrix of L6E9 skeletal myoblasts and CF infected with *T. cruzi* and treated with TGF-β per 48 h was evaluated by indirect immunofluorescence. *T. cruzi* infection resulted in a reduction in general FN expression in L6E9 skeletal myoblasts (Figure 4B,C,E,F,H,I), while uninfected controls displayed widespread distribution of FN fibrils (Figure 4A,D,G). On the other hand, CF displayed a general intensification of the FN staining, which was localized in the uninfected cells of the cultures, when compared to controls (Figure 5A,D,G) with less or no FN fibrils distributed over highly infected cells (Figure 5B,C,E,F,H,I). Highly infected L6E9 skeletal myoblasts (Figure 4E,F,H,I) and CF (Figure 5E,F,H,I) cultures treated with TGF-β (1 and 10 ng/mL) showed a general increase in FN fibrils in the culture compared with the untreated controls (Figure 4A–C and Figure 5A–C). However, the FN increase was localized in adjacent non-infected cells, while highly infected cells displayed a reduction in FN distribution, even after addition of high doses of TGF-β (1–10 ng/mL (Figure 4E,F,H,I and Figure 5E,F,H,I) compared to untreated controls. In both cell types, the remaining FN expression was redistributed, being seen along the borders of the infected cells instead of as the widespread fibrils observed in uninfected controls ( Figure 4 and Figure 5). Measurement of FN staining area through image processing with Image J (Figure 4J and Figure 5J) confirmed these observations. L6E9 showed a decrease of 29% on FN fibrils after *T. cruzi* infection, while CF increased 1.5-fold from control in infected cultures. With addition of TGF-β, the area occupied by FN increased 3.2-fold (1 ng/mL) and 3.5-fold (10 ng/mL) for L6E9 (Figure 4J), while CF cultures increased 2.8-fold (1 ng/mL) and 2.3-fold (10 ng/mL) (Figure 5J). *T. cruzi* infection prevented FN stimulation by TGF-β in both cell types, with L6E9 skeletal myoblasts showing a reduction in FN fibril thickness of 72% for 1 ng/mL and 79% for 10 ng/mL when compared to uninfected treated peers (Figure 4J). CF also presented low response to TGF-β, with the FN area remaining similar to control after TGF-β treatment, representing decreases of 37% (1 ng/mL) and 21% (10 ng/mL) when compared to treated and uninfected controls (Figure 5J). Even though we could not detect the same level of TGF-β stimulation of FN through image processing revealed by Western blot, since our measurements took in account only the area occupied by FN fibrils and not the intensity of staining, the modulation patterns were similar between the two approaches and confirmed the biological phenomenon. The treatment of cardiac fibroblast cultures with TGF-β did not impact *T. cruzi* levels of infection, as there were no significant differences in the percentage of infected cells or at the number of intracellular amastigotes (Supplemental Material, Figure S3).

We also performed staining of actin cytoskeleton in cardiac fibroblast cultures. *T. cruzi* infection resulted in myofibrillar breakdown, which impacted FN network organization, even after TGF-β treatment (Supplementary Material, Figure S4).

### 2.5. Signaling Pathways Involved in ECM Modulation Triggered by T. cruzi Cardiac Fibroblast Infection

**Figure 6:**
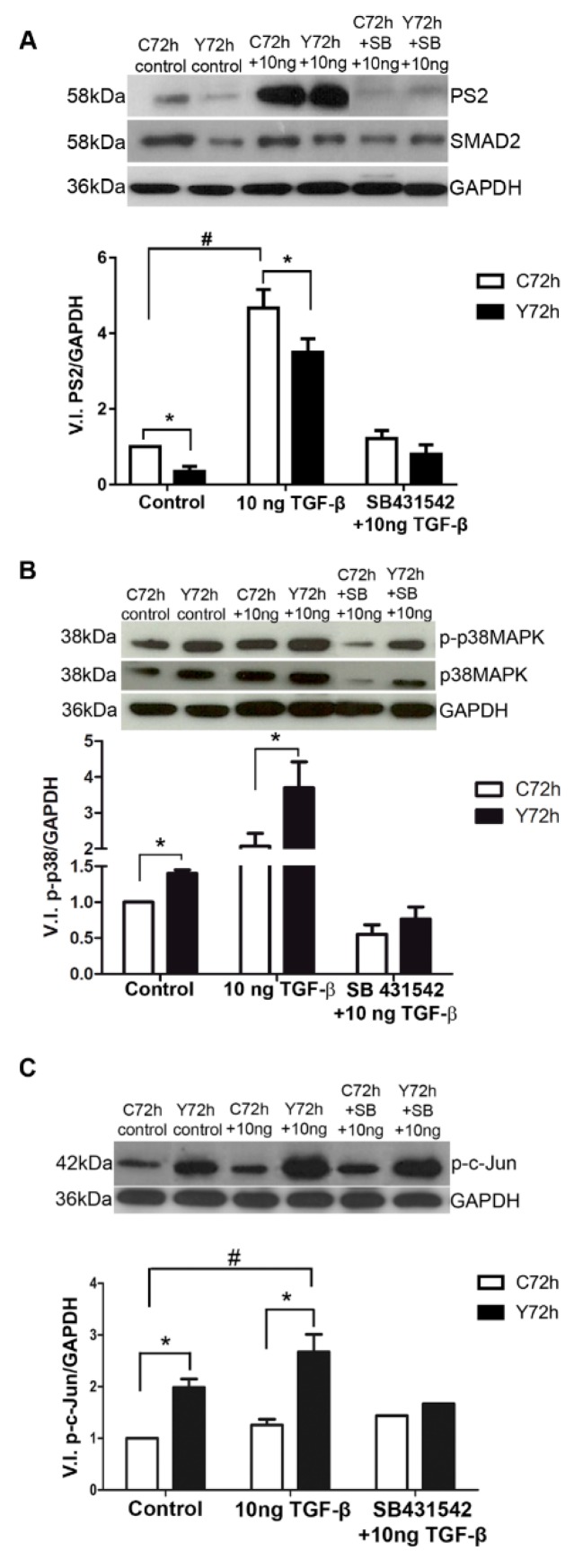
PS2, phospho-p38 MAPK, and phospho-c-Jun detection in normal and infected cardiac fibroblasts with *T. cruzi*. (**A**) PS2 detection. Normal CF showed an increase of SMAD2 phosphorylation when treated with 10 ng/mL of TGF-β. Infected CF were unable to trigger the classical signaling pathway, even when treated with 10 ng/mL, compared to the uninfected pair. The SB431542 inhibitor prevented SMAD2 phosphorylation in CF cultures after stimulation with TGF-β. (**B**) Phosphorylated p38 MAPK detection. In normal CF treated with 10 ng/mL of TGF-β, an increase of p38 MAPK phosphorylation detection was observed. *T. cruzi* infection did not result in significant differences in p38 MAPK phosphorylation, although there was a tendency to increase in this signaling pathway. After addition of 10 ng/mL of TGF-β, the signaling pathway was triggered in CF. The inhibitor SB431542 prevented the p38 MAPK phosphorylation in both normal and infected CF treated with 10 ng/mL TGF-β. (**C**) Phosphorylated c-Jun detection. Note that infection by *T. cruzi* led to an increase in the c-Jun signaling pathway. The addition of TGF-β in normal cultures resulted in a 25% increase in c-Jun detection. The increase triggered by the infection remained and was enhanced by treatment with TGF-β. In all panels, white bars correspond to control, uninfected cultures (C72h), while black bars depict levels of *T. cruzi* (Y strain) infected cultures (Y72h). * *p* ≤ 0.05 compared their control pairs; # *p* ≤ 0.05 compared to untreated, uninfected controls *N* = 3.

The association of fibroblasts, the effector cells of fibrosis, with relative down-regulation of FN expression in *T. cruzi*-infected cells after TGF-β stimulation led us to evaluate (in detail) the regulation of the signaling pathways involved in ECM modulation. We analyzed the activation profiles of PS2, phospho-p38 MAPK, and phosho-c-Jun signaling pathways in uninfected and *T. cruzi*-infected CFs (72 h) subjected (or not) to stimulation with 10 ng/mL of TGF-β. A 59% reduction in PS2 activation was evidenced in the infected CF cultures when compared to their uninfected counterparts (Figure 6A). Both uninfected and *T. cruzi*-infected CF cultures were stimulated by the cytokine treatment. Addition of exogenous TGF-β induced a significant 4.6-fold increase in PS2 activation of *T. cruzi*-infected CF cultures compared to unstimulated infected cultures (Figure 6A). However, a 28% reduction in the activation of the SMAD2 pathway was evidenced when compared to PS2 phosphorylation levels of the uninfected and TGF-β stimulated culture (Figure 6A). As expected, an inhibition of PS2 activation was revealed after treatment of cultures with the specific pharmacological inhibitor SB431542 (ALK5 signaling inhibitor), reaching levels similar to controls (Figure 6A). 

Interestingly, high activation of p38 MAPK was revealed in CFs infected with *T. cruzi* as compared to uninfected cultures, achieving a significant 54% increase in the activation of this pathway. The addition of 10 ng/mL of TGF-β induced a rise in the p38 MAPK phosphorylation in uninfected and infected CF cultures, but its activation was 78% higher in infected cultures. SB431542 treatment prior to TGF-β stimulation led to a reduction of p38 MAPK phosphorylation in both conditions (Figure 6B).

Up-regulation of c-Jun signaling pathway was also induced by *T. cruzi* infection, showing a significant 1.98-fold increase of c-Jun phosphorylation compared to uninfected CFs (Figure 6C). Although 25% activation of c-Jun pathway was observed in stimulated uninfected cells, the increase in phosphorylated c-Jun levels in stimulated *T. cruzi*-infected CFs exceeded this value by 35%. SB431542 also prevented c-Jun phosphorylation in uninfected and *T. cruzi*-infected CF cultures. 

**Figure 7:**
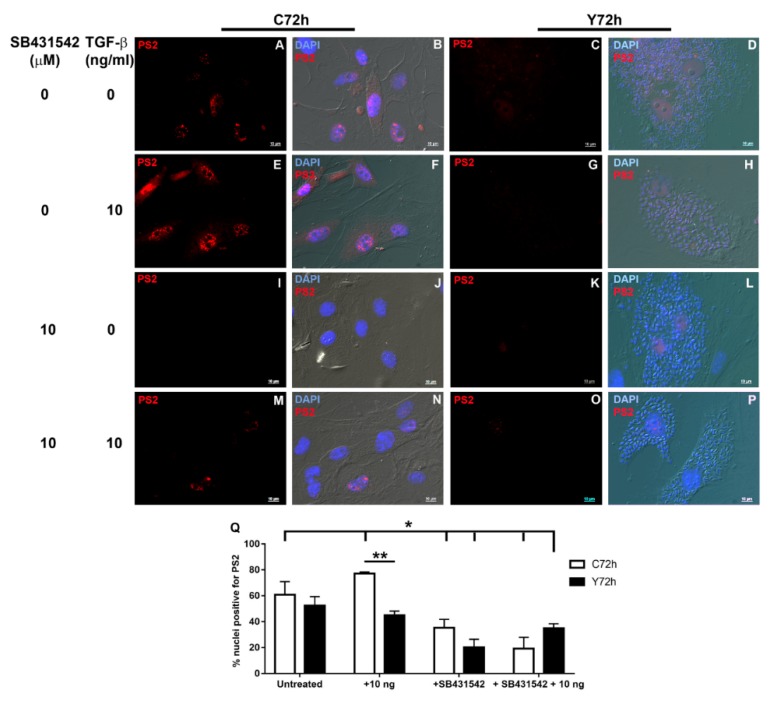
Phosphorylated SMAD2 detection in *Trypanosoma cruzi* infected cardiac fibroblasts. PS2 staining is represented in red (**A**,**C**,**E**,**G**,**I**,**K**,**M**,**O**), while cell nuclei are depicted in blue after DAPI staining together with DIC (**B**,**D**,**F**,**H**,**J**,**L**,**N**,**P**). (**A**,**B**) Control, uninfected cultures (C72h), showing constitutive PS2 expression; (**C**,**D**) *T. cruzi* infection (72h- Y72h) reduced PS2 detection (Y72h); (**E**,**F**) TGF-β treatment stimulated PS2 in uninfected cultures; (**G**,**H**) Infection with *T. cruzi* prevented PS2 signaling in CF; SB431542 inhibited PS2 detection in uninfected (**I**,**J**) and *T. cruzi* infected (**K**,**L**) CF cultures; (**M**,**N**) SB431542 prevented TGF-β stimulation of PS2; (**O**,**P**) *T. cruzi* infection and SB431542 treatment resulted in low detection of PS2 in CF. (**Q**) Quantification of percentage of nuclei positive for PS2. * *p* ≤ 0.05 vs. untreated, uninfected control; ** *p* ≤ 0.05 vs. C72h + 10 ng/mL TGF-β. Bar = 10 μm.

To evaluate if *T. cruzi* could modulate phosphorylated SMAD2 (PS2) transduction to the nucleus of infected cells, we treated normal and infected cardiac fibroblasts with TGF-β, pre-treated or not with ALK5 inhibitor SB431542, and analyzed PS2 localization by immunofluorescence. Our data showed that the uninfected cells presented constitutive PS2 in nuclei and cytoplasm (Figure 7A,B). Addition of TGF-β induced SMAD2 phosphorylation with intense staining in the nuclei (Figure 7E,F), and there was a 50% increase in the number of nuclei positive for PS2 (Figure 7Q). Addition of SB431542 reduced constitutive PS2 expression (Figure 7I,J) with a 26% decrease in PS2 positive nuclei (Figure 7Q). The inhibitor also prevented TGF-β stimulus of PS2 (Figure 7M,N), corresponding to 66% inhibition of PS2 activation in the nucleus when compared to TGF-β treated CF (Figure 7Q). *T. cruzi* infection of cardiac fibroblasts reduced PS2 detection in host cell nuclei (Figure 7C,D) with a decrease of 10% of nuclei PS2 localization. The presence of intracellular parasites resulted in a significant inhibition of PS2 activation after TGF-β treatment (Figure 7G,H) with a 45% reduction of nuclear localization of PS2 when compared to uninfected TGF-β treated controls (Figure 7Q). *T. cruzi* infection had a synergic effect with SB431542 in CF with low detection of PS2 (Figure 7K,L) and a reduction of 48% of PS2 positive nuclei when compared to uninfected SB431542 treated controls (Figure 7Q). Pre-treatment of infected cultures with ALK5 inhibitor prior to addition of TGF-β also resulted in low PS2 detection in CF cultures (Figure 7O,P) and induced a 54% inhibition in the number of PS2 positive nuclei when compared to TGF-β treated and uninfected peers (Figure 7Q).

### 2.6. Induction of Cardiac Fibroblast Proliferation by T. cruzi Infection and TGF-β Stimulation

**Figure 8:**
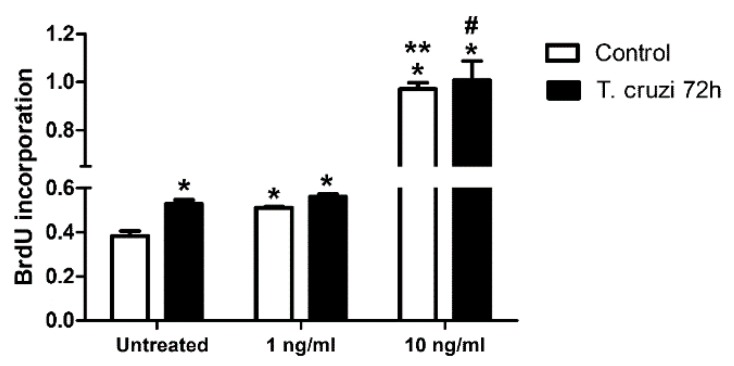
Cardiac fibroblast proliferation treated with TGF-β. Cardiac fibroblasts treated with different concentrations of TGF-β (1 and 10 ng/mL) showed a high proliferation in a dose dependent manner when compared with CF untreated. * *p* ≤ 0.05 vs. untreated, uninfected control; ** *p* ≤ 0.05 vs. control + 1 ng/mL TGF-β; # *p* ≤ 0.05 vs. *T. cruzi* 72 h + 1 ng/mL TGF-β. *N* = 3.

To evaluate whether *T. cruzi* infection and TGF-β played a role in the proliferation of CF, these cells were infected with *T. cruzi* and treated for 48 h with different concentrations of TGF-β (1–10 ng/mL). Interestingly, *T. cruzi* infection augmented CF proliferation by 37% as compared to uninfected cells. Both uninfected and *T. cruzi*-infected cultures showed similar responses in the induction of CFs proliferation after stimulation with TGF-β, and 2.5-fold higher levels in cell proliferation profiles were revealed from the stimulation of 10 ng/mL TGF-β in both uninfected and *T. cruzi*-infected cultures (Figure 8).

## 3. Discussion

Cardiac fibrosis is a major feature of cardiomyopathy, increasing the deposition and the accumulation of extracellular matrix proteins in the myocardium. TGF-β is a pro-fibrogenic cytokine and exerts a significant role in the heart as a regulator of components of ECM and is implicated in the genesis of cardiac fibrosis in Chagas disease [14]. 

In this study, FN expression and its distribution in ECM were analyzed in normal and *T. cruzi*-infected CF and L6E9 skeletal myoblasts treated with TGF-β (1–10 ng/mL). Also, the roles of classical and alternative TGF-β signaling pathways in the FN modulation in ECM in CF were investigated. Our data revealed that CF and L6E9 skeletal myoblasts showed an increase of FN starting in low concentrations of TGF-β (1–10 ng/mL). In contrast, normal cardiomyocytes displayed ECM stimulus only when treated with 10 ng/mL, similar to results previously reported by our group [33]. The differences observed in ECM expression in the different cell types could be explained by distinctive expression of TGF-β receptors, such as endoglin, an auxiliary type III TGF-β receptor that controls ECM expression in response to TGF-β [39]. Skeletal muscle cells do not have endoglin on their surface [40]. In CF, endoglin is constitutively expressed and is critical for TGF-β1 signaling, also modulating type I collagen synthesis in these cells [41]. Expression of endoglins, which is poorly understood in cardiomyocytes, occurs during the formation of the heart in the embryonic development, mainly in endothelial cells [42].

L6E9 skeletal myoblasts infected with *T. cruzi* showed a reduction of FN expression, even after treatment with higher doses of TGF-β. In contrast, in CF, the overall measurement of the culture by Western blot showed an increase of FN, while immunofluorescence demonstrated that highly infected cells presented a redistribution of FN to the borders of the cell. In both cases, the parasite may have been directly modulating synthesis, secretion, and organization of matrix proteins. In the case of CF, *T. cruzi* seemed to be preventing the cell response to exogenous TGF-β stimulus. Evidence from the literature supports this idea, since a secreted or released factor from *T. cruzi* is capable of repressing connective tissue growth factor (CTGF) / cellular communication network factor 2 (CCN2) expression in response to TGF-β in dermal fibroblasts, an effect also observed in the down-regulation of fibrogenic genes after infection [43]. The localized FN reduction and matrix delocalization in *T. cruzi*- infected L6E9 myoblasts and CF could also be associated with the actin cytoskeleton breakdown caused by the infection, which prevents both the anchoring of FN to integrins on the cell surface and the organization of the FN matrix [44,45]. Our results support this hypothesis, since *T. cruzi* infection disrupted the actin cytoskeleton of CF. Previous work from our group also showed a disorganization of the TGF-β receptor type II cell surface distribution induced by *T. cruzi* in cardiac fibroblast-containing primary cardiomyocyte cultures, which is associated with a lower global PS2 signaling response to exogenous TGF-β [46]. Furthermore, *T. cruzi* infection induces a significant reduction in the global levels of mRNA in the cytoplasm of host cell concomitant with the amastigote proliferation in primary cardiomyocyte cultures that also contain CF [47], suggesting that the intracellular multiplication of *T. cruzi* can affect the mRNA stability in the host, an effect that could result in reduced levels of protein synthesis. 

In cardiac cultures infected with *T. cruzi*, different cytokines and chemokines such as TNF-α, IL-1β, and iNOS are secreted in response of the infection [48]. The increase of FN expression in CF infected cultures suggests that the cellular stress against infection causes a release of cytokines such as TNF-α, and that adjacent uninfected cells of the infected culture receive cytokine stimulus together with TGF-β, potentiating and modulating the synthesis and the release of FN in the ECM. It was observed that, in cardiac fibrosis, TNF-α may be involved in excessive accumulation of ECM in the myocardium [49]. Furthermore, mixed cultures containing cardiomyocytes and CF showed an increase in FN in response to stimulation with TNF-α [33].

Our results demonstrated that CF and skeletal L6E9 myoblasts showed higher SMAD2 phosphorylation than CM after TGF-β stimulation. However, p38 MAPK levels were higher in CM and skeletal L6E9 myoblasts when compared with CF. The differences in signaling responses could have been caused by differences in TGF-β receptor expression between the different cell types, including endoglins, as discussed above. In addition, in cardiac tissue, the p38 MAPK signaling pathway is a dominant response to injury, leading to cardiomyocyte hypertrophy through further activation of MKK3 and MKK6 [50]. Our data showed that p38 MAPK detection was higher in cardiomyocytes than CF, suggesting that the p38 MAPK pathway may have resulted in hypertrophy rather than fibrosis in cardiomyocytes.

Our findings demonstrated that PS2 levels were reduced in *T. cruzi*-infected CF cultures after the addition of TGF-β (10 ng/mL) when compared to uninfected controls. We also demonstrated that *T. cruzi* infection significantly prevented TGF-β stimulus of PS2 localization in CF nuclei. The presence of the intracellular parasite in CF seemed to be modulating the classical signaling pathway and preventing the signal transduction to the nucleus. Studies in cardiomyocyte cultures have shown that *T. cruzi* uptakes endogenous TGF-β for multiplication and development in the host cell, using it for its own cycle [25]. Also, the secreted factor from *T. cruzi* that inhibits TGF-β response in dermal fibroblasts [43] might be disrupting SMAD signaling in CF. 

We demonstrated an increase of p38 MAPK phosphorylation in CF after *T. cruzi* infection and treatment with TGF-β. The p38 MAPK phosphorylation can modulate the deposition of ECM proteins in infected CF and might be a candidate for intervention against Chagas disease fibrosis. In *T. cruzi*-infected macrophage cultures, the exogenous addition of cruzipain, the most abundant cysteine protease of *T. cruzi*, and the JNK pathway inhibitor, SP600125, induces triggering and amplification of the p38 MAPK signal in this cell type, favoring survival and amplification of the parasite in macrophages [51]. In other models of cardiomyopathy, p38 MAPK also was proven to be a critical pathway for fibrosis, since conditional knock-out of the p38 MAPK gene, *Map14k*, in cardiac fibroblasts blocked myofibroblast differentiation after ischemic injury and led to reduced heart fibrosis [52]. 

Our data revealed that *T. cruzi* infection increased c-Jun phosphorylation in CF. When TGF-β bound to it specific receptor, a rapid c-Jun activation occurred depending on the cell type, leading to SMAD3 phosphorylation. Therefore, c-Jun signaling may have increased the classical signaling pathway through SMAD3 phosphorylation [53,54]. In *T. cruzi*-infected macrophage cultures, an increase in c-Jun phosphorylation was similarly observed [51]. Likewise, significant up-regulation of c-Jun was detected in early *T. cruzi* infection of primary human colonic epithelial cells by phosphoproteomics and Western blot [55]. In addition, primary cultures of cardiac myocytes displayed an increase in nuclear translocation of JunB up to 2 h after *T. cruzi* infection [56]. These data demonstrated that the parasite has an important role in the activation of c-Jun in different cell types and also in CF cultures, where an increase in c-Jun phosphorylation triggered by infection was observed independent of TGF-β treatment, suggesting c-Jun as a candidate target for intervention against Chagas disease fibrosis in the heart.

To investigate if FN increase resulting from *T. cruzi* infection was caused by CF proliferation stimulated by the parasite, we measured 5-bromo-2′-deoxiuridin (BrdU) incorporation in *T. cruzi*-infected CF cultures treated with different concentrations of TGF-β. Our results showed that *T. cruzi* infection and TGF-β promoted the proliferation of cardiac fibroblasts, with TGF-β response being observed in a dose-dependent manner, an effect consistent with fibrosis development in the myocardium in CD. Different sources of evidence show that *T. cruzi*-derived molecules can modulate fibroblast proliferation. Conditioned media from *T. cruzi*-infected cultures and amastigote extract stimulated [^3^H]thymidine incorporation in human dermal fibroblasts, although the authors were not able to identify which parasite molecule was responsible for this process [57]. More recently, recombinant *T. cruzi* calreticulin (TcCRT) was shown to induce fibroblast migration in scratch plate assays and increase proliferation of human dermal fibroblasts three orders of magnitude more efficiently than the recombinant human calreticulin [58]. Thus, *T. cruzi* can potentially modulate cardiac fibroblast proliferation either by direct infection or through a paracrine effect of infected cells over uninfected cells, or possibly via the remaining antigens of dead parasites in the Chagas patient’s cardiac tissue [59] acting through *T. cruzi* calreticulin. Cardiac fibroblast proliferation can be targeted for treatment, since tetrandrine, a drug used for cancer treatment, can inhibit the proliferation of cardiac fibroblasts induced by TGF-β [60] and potentially could be repurposed for treatment of cardiac fibrosis in Chagas Disease. 

Altogether, our studies open new perspectives to understand the regulatory mechanisms of TGF-β in FN matrix in cardiomyocytes, cardiac fibroblasts, and L6E9 myoblasts and uncover new therapeutic targets against cardiac fibrosis in Chagas Disease.

## 4. Materials and Methods

### 4.1. Primary Cardiomyocyte Culture

Primary cultures of cardiomyocytes and cardiac fibroblasts were performed using 18 day old mouse embryos as previously described [61]. Cardiac fragments were dissociated in phosphate buffered saline (PBS; 137 mM NaCl, 2.7 mM KCl, 0.88 mM KH_2_PO_4_, 6.4 mM Na_2_HPO_4_, pH7.2) containing 0.025% trypsin and 0.01% collagenase (Worthington Co., Lakewood, NJ, USA) and plated into a 24-well plate (10^5^ cells/mL) containing glass coverslips coated with 0.01% gelatin. Although cardiomyocytes constitute approximately 80% of the total cells, the culture still displayed myoblasts and fibroblasts. Cultures were cultivated in Dulbecco’s modified Eagle medium (DMEM; Sigma Chemical Co, St. Louis, MO, USA) supplemented with 10% fetal bovine serum (FBS; Sigma), 2.5 mM CaCl_2_, 1 mM L-glutamine, and 2% chicken embryo extract and kept at 37 °C in an atmosphere of 5% CO_2_.

### 4.2. Cardiac Fibroblasts and Skeletal Muscle Myoblasts Culture

CF cultures were purified from successive dissociations of mouse cardiac muscle cells primary cultures. Cells seeded in 25 cm^2^ culture flasks were grown in DMEM supplemented with 10% FBS, 2% chicken embryo extract, 2.5 mM CaCl_2_, 1 mM L-glutamine, and antibiotics and kept at 37 °C in 5% CO_2_ atmosphere. Fibroblast purification was carried out by the differential plating method and successive subculture based on the slower adhesion of cardiomyocytes to the substrate and its susceptibility to sequential enzyme activity, respectively. Rat skeletal muscle myoblasts from L6E9 lineage were maintained in DMEM supplemented with 10% FBS, 1 mM L-glutamine. The cells were subcultured by the dissociation of confluent cultures with a solution of 0.025% trypsin and 0.01% EDTA in PBS. After dissociation, the isolated cells were counted and seeded at a density of 5 × 10^4^ cells/well and 5 × 10^5^ cells/dish in 24-well plates and 60 mm dishes, respectively.

### 4.3. Parasites and Cell Culture Infection

Trypomastigotes of *T. cruzi*, Y strain, derived from Vero culture were used. Cardiac muscle cells, CF, and skeletal myoblasts L6E9 were infected at a multiplicity of infection of 10 parasites/host cell (10:1) after 24 h of cultivation. The infection was interrupted after 72 h.

### 4.4. Treatment of Cardiomyocytes, Cardiac Fibroblasts, and Skeletal Myoblasts L6E9 with TGF-β

Normal and 24 h infected cultures were washed with Ringer’s solution to remove the FBS contained in the nutritive medium. The treatment was performed with recombinant TGF-β (R&D Systems, Minneapolis, MN, USA) diluted in DMEM supplemented with 0.1% FBS, 2.5 mM CaCl_2_, and 2% L-glutamine. TGF-β was added at concentrations of 1 and 10 ng/mL after 24 h of cultivation. The cells were fixed after 48 h of TGF-β treatment and 72 h of *T. cruzi* infection. For analysis of signaling pathways, the treatment with recombinant TGF-β was performed for 1 h in the same concentrations in normal and *T. cruzi*-infected cultures (72 h).

### 4.5. Indirect Immunofluorescence

Cardiomyocytes, CF, and skeletal myoblasts L6E9 treated or not with TGF-β were fixed for 5 min at room temperature with 4% paraformaldehyde (PFA) in PBS followed by washing in PBS. To block nonspecific reactions, monolayers were washed (three times for 20 min) with PBS containing 4% bovine serum albumin (BSA). The cells were then incubated for 18 h at 4 °C with anti-fibronectin antibody (1:400; Sigma Chemical Co.) or anti-phosphorylated SMAD2 antibody (1:200; Cell Signaling, Danvers, MA, USA). After successive washes in PBS, the cultures were incubated for 1 h at 37 °C with secondary anti-rabbit antibody TRICT-conjugated (1:200; Sigma). Phalloidin-FITC (4 μg/mL, Sigma Chemical Co.) was added together with the secondary antibodies to display actin microfilaments. For visualization of the nucleus, cells were stained with DAPI (DNA dye), and then the coverslips were mounted in 2.5% 1’4-Diazabicyclo-(2.2.2)-octane (DABCO, Sigma Chemical Co.) in PBS/50% glycerol and sealed with nail polish. As negative controls, the primary antibodies were omitted, and no unspecific reaction was visualized (Supplemental Material, Figure S5). The images were acquired at the confocal laser scanning microscope LSM 510 Meta (Zeiss, Oberkochen, BW, Germany) or Bx512 Fluoview 500 (Olympus, Shinjuku, Tokyo, Japan), where differential interference contrast (DIC) images showed localization of intracellular parasites. The fraction of total area of the field occupied by FN staining was measured using FIJI software [62]. The raw images were segmented by color thresholding defined in images from control, untreated, and uninfected samples. After generation of binary images, the percentage of total area of the field covered by FN staining was measured. Immunofluorescence for PS2 was observed and acquired at an Axio Image M2 equipped with Apotome system (Olympus).

### 4.6. Protein Extraction

Cardiomyocytes, CF, and L6E9 skeletal myoblasts treated or not with TGF-β were washed 3 times with cold PBS on ice. Then, the cells were scraped in lysis buffer (50 mM Tris, 150 mM NaCl, 1% Triton X-100, pH 8.0) containing phosphatase and protease inhibitors (PhosStop Roche Diagnostics, Basel, Switzerland), 1 mM EGTA, 1 mg/mL pepstatin, 100 mg/mL PMSF, 1 μg/mL aprotinin and 2 mg/mL leupeptin. After the lysis, electrophoresis sample buffer 5X was added (0.3 M Tris, 10% SDS, 0.125% Bromophenol Blue, 25% β-mercaptoethanol, and 50% glycerol), and the samples were heated to 100 °C for 5 min in a dry bath. After the samples cooled down to room temperature, the samples were stored at –20 °C. Before adding the sample buffer, an aliquot of each sample was separated to quantify the total protein amount using the Folin–Lowry method.

### 4.7. Western Blot

After the determination of protein concentration, 10 µg or 20 µg of total protein extracts obtained from cardiomyocytes, CF, and skeletal myoblasts L6E9 normal and infected and treated or not with TGF-β were subjected to electrophoresis on 10% and 12% of polyacrylamide gel containing SDS (SDS-PAGE). The electrophoretic separated proteins were transferred to nitrocellulose membranes and incubated for 1 h at 4 °C with blocking buffer consisting of 25 mM Tris, 150 mM NaCl, 0.05% Tween 20 (TBST), 5% non-fat dry milk (Molico), and 0.1% Tween 20. After blocking, the membranes were incubated with anti-fibronectin antibody (1:5000; Sigma Chemical Co.), anti-phosphorylated SMAD2 (1:2000; Millipore, Burlington, MA, USA ), anti-phosphorylated p38 (1:1000; Cell Signaling), and anti-phosphorylated c-Jun (1:500; Millipore) for 18 h at 4 °C. Anti-GAPDH (1:50,000; RDI Fitzgerald, Acton, MA, USA) was used as internal control. The membranes were then washed with TBST and incubated for 1 h at room temperature with anti-rabbit or anti-mouse peroxidase conjugate (Thermo Scientific Pierce Protein Biology, Waltham, MA, USA ) diluted 1:20,000 and 1:30.000, respectively, in blocking buffer. The membranes were washed, and the peroxidase was revealed by chemiluminescence using the Super Signal West Pico (Thermo Scientific Pierce Protein Biology) kit. Densitometry of the resulting bands was performed with the Image J program (http://rsbweb.nih.gov/ij/).

### 4.8. Cardiac Fibroblasts Proliferation

CFs plated in a density of 1.5 × 10^4^ cells per well in 96-well plates were infected with *T. cruzi* (Y strain, 10 parasites/host cell), and after 24 h of infection, the cultures were treated with 1, 5, and 10 ng/mL of TGF-β per 48 h. Measurement of proliferation of CFs was performed using BrdU Cell Proliferation Kit (Millipore) according to manufacturer’s instructions. Briefly, after 48 h of treatment with TGF- β and 72 h of *T. cruzi* infection, the cultures were incubated with 20 µg/mL of 5-bromo-2′-deoxiuridin (BrdU), allowing its incorporation in proliferating cells for 2 h. Cells were fixed, and the DNA was denatured with the solution provided by the kit for 30 min at room temperature. After washing, the BrdU-labeled DNA was detected by the monoclonal anti-BrdU antibody for 1 h at room temperature. The plate was washed, and the antigen–antibody complex was revealed by addition of peroxidase-conjugate goat anti-mouse IgG, antibody, and 3,3′,5,5′-Tetramethylbenzidine (TMB) as peroxidase substrate. The reaction was stopped with acid Stop Solution, and the colorimetric reaction was read in an M2 Spectramax Plate Reader (Molecular Devices, San Jose, CA, USA) at λ450 nm.

### 4.9. Statistical Analysis

Student’s *t*-test was used for comparison of experimental data from FN immunostaining area measurements, densitometry of Western blotting bands, and quantification of PS2 positive nuclei. Values were considered statistically significant when *p* ≤ 0.05.

## Supplementary Materials

Supplementary materials can be found at https://www.mdpi.com/1422-0067/20/19/4836/s1.
