# Supplementary material for: Differential Role of TGF-β in Extracellular Matrix Regulation During Trypanosoma cruzi-Host Cell Interaction
Source: Int J Mol Sci. 2019 Sep 29;20(19):4836. doi: 10.3390/ijms20194836 (PMC6801917; doi:10.3390/ijms20194836)
Supplement: Supplementary file 1 [file ijms-20-04836-s001.pdf]

International Journal of *Molecular Sciences*

MDPI

Supplementary Materials

# Differential role of TGF-β in extracellular matrix regulation during *Trypanosoma cruzi* - host cell interaction

**Tatiana Araújo Silva, Luis Felipe de Carvalho Ferreira, Mirian Claudia de Souza Pereira and Claudia Magalhães Calvet*.**

Cellular Ultrastructure Laboratory, Oswaldo Cruz Institute, FIOCRUZ, Rio de Janeiro, RJ, 21040-360, Brazil

*Corresponding author: Dr Claudia M. Calvet, Laboratório de Ultraestrutura Celular, Instituto Oswaldo Cruz, FIOCRUZ, Av. Brasil 4365, Manguinhos, 21040-362 Rio de Janeiro, RJ, Brazil, e-mail: cmcalvet@ioc.fiocruz.br

1. **Characterization of cardiac fibroblasts.**

Cardiac fibroblasts have a larger cell nucleus when compared to cardiomyocytes (CM), and this morphological trait is a distinctive feature of this cell type. Initially, CF purification obtained from CM cultures was evaluated only by morphology. CF obtained in the third dissociation of CM cultures were infected with *T. cruzi* and treated with TGF-β for 48 hours and were subjected to Western blot to analyze the FN expression under these conditions. Our data revealed an increase in FN expression only with 10 ng/ml TGF-β. Furthermore, third dissociation from CM cultures had reduced FN expression compared to normal control even after the addition of TGF-β. The addition of inhibitors SB431542 and SB203580 (SMADS pathway and p38 MAPK inhibitor respectively) in this culture led to inhibition of FN stimulation by TGF-β, since in infected cultures treated with 10 ng/ml TGF-β (48h) reduced FN expression after pre-treatment with inhibitors signaling (S1A) was seen. The data obtained in the third dissociation from CM culture extracts with the FN expression is very similar to that of cardiomyocytes [1], suggesting that the purification process of the CF needed adjustments and that FN response could not be displayed properly when CF is present in CM culture, suggesting that morphological analysis under the microscope do not guarantee good purification of CF.

Thus, to assess the degree of CF purification from CM cultures, desmin, a specific cytoskeletal protein of cardiomyocytes, was evaluated in CF extracts after different dissociations. CF extracts obtained in the third and fourth dissociation revealed that cardiomyocytes are still present in CF culture, since the extracts also had high expression of desmin, demonstrating that these dissociations are not viable for conducting experimental tests. Pure CF was obtained only from the fifth dissociation from CM culture in the absence of cardiomyocytes, with desmin undetectable in this culture only after the 5th passage (S1B).

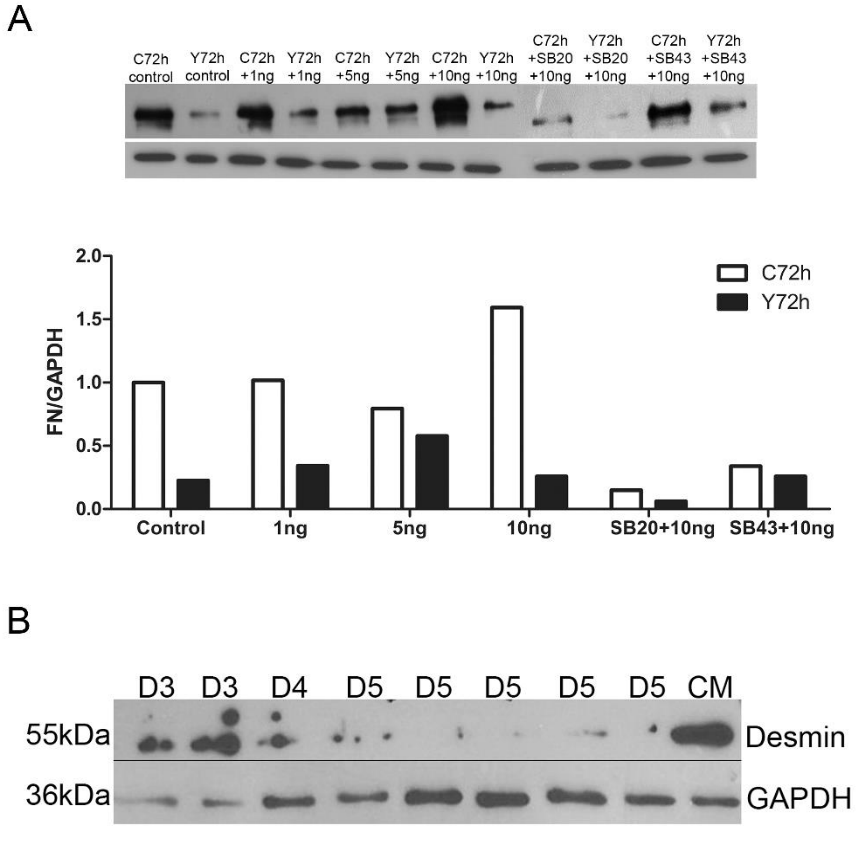


**Figure S1 - FN expression in cells obtained in the third CM culture passage. (A) Control cultures show FN stimulation only with the treatment with 10 ng/ml TGF-β (48h).** Doses of 1ng/ml and

5ng/ml did not induce increase of FN expression. *T. cruzi* infection leads to a reduction in FN expression after addition of TGF-β. Inhibitory action of SMADs and p38 MAPK pathway prevents increase in FN expression even after stimulation with 10 ng/ml. These results obtained with cell extracts after third CM culture passage are similar to previously published data in cardiomyocyte cultures under the same conditions. **(B) Characterization of the culture of heart fibroblasts**. Rate of CF purification from cardiac culture. The degree of CF purification was evaluated by detection of desmin in cell extracts obtained in the 3rd, 4th and 5$^{th}$ dissociation. CF purification was obtained from the fifth dissociation, as shown by the absence of desmin.

## 2. TGF-β induced with *T. cruzi* infection contributes to FN upregulation in cardiac fibroblasts

To evaluate the participation of TGF-β directly induced by *T. cruzi* in FN stimulus, we treated 24h infected cultures with SB431542 (10μM) and analyzed FN expression after 72h of infection. The inhibition of ALK5 prevented the increase of FN expression resulting from *T. cruzi* infection (Fig. S4), suggesting that TGF-β stimulated by the parasite can be participating in the induction of FN synthesis.

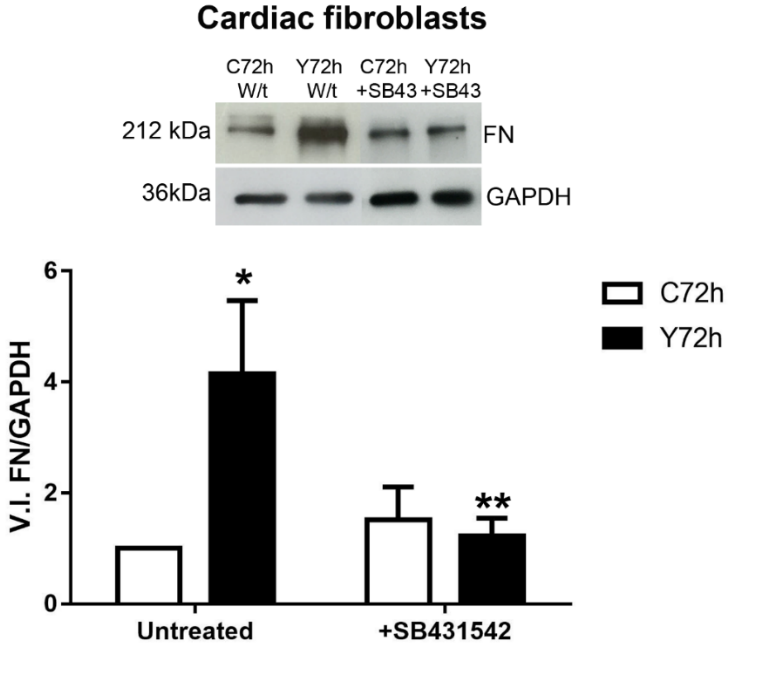


**Figure S2- FN expression in cardiac fibroblasts after treatment with signaling inhibitors.** *Trypanosoma cruzi* infection (Y72h) in cardiac fibroblasts results in an increase in FN levels detected by western blot when compared to uninfected controls (C72h). Inhibition of SMAD signaling pathways activation by SB431542 prevented the FN stimulus triggered by *T. cruzi* infection in cardiac fibroblasts. White bars correspond to control, uninfected cultures (C72h), while black bars depict levels of *T. cruzi* (Y strain) infected cultures (Y72h). * $p \leq 0.05$ vs. untreated C72h; ** $p \leq 0.05$ vs. untreated Y72h. N=3

## 3. Treatment of cardiac fibroblast cultures with TGF-β does not impact *T. cruzi* levels of infection

The profile of *Trypanosoma cruzi* infection was quantified in cardiac fibroblasts by manual counting of the number of total cells, infected cells and intracellular parasites, allowing the calculation of percentage of infection in the culture, as well as the number of intracellular amastigotes in infected cells. Untreated cultures showed a high degree of infection after 72h of interaction, with 80.5 ± 17.3%

of infected cells on the culture, with 137± 29 parasites per infected cell. The treatment of the cultures with TGF-β did not affect infection levels, with cultures treated with TGF-β displaying 90 ± 15% infection and 138.4 ± 62 parasites per infected cell (1 ng/ml) and 90.3 + 8.7 % infection and 144 ± 24 parasites per infected cell (10 ng/ml).

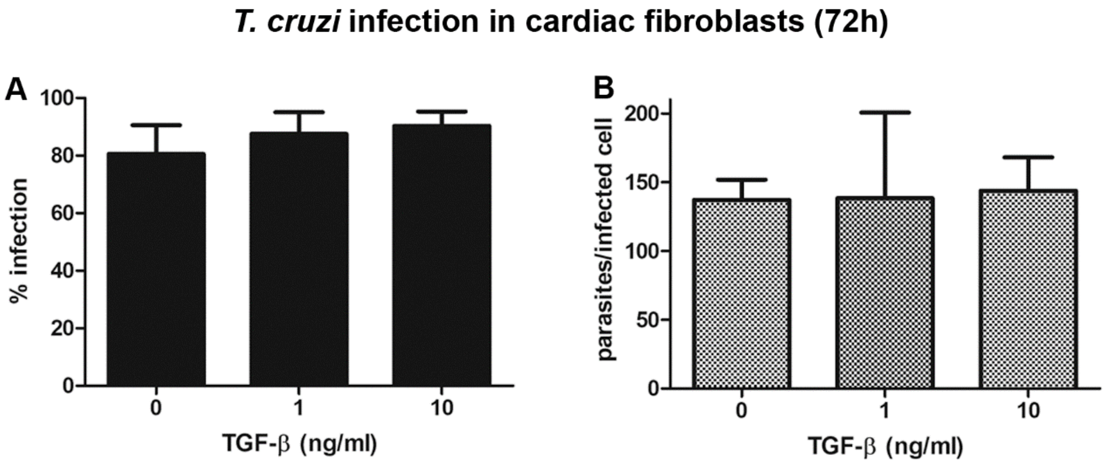


**Figure S3- Quantification of *T. cruzi* infection levels in cardiac fibroblasts.** (A) Percentage of infected cardiac fibroblasts after TGF-β treatment; (B) Number of intracellular amastigotes per infected cell with addition of TGF-β.

### 4. *Trypanosoma cruzi* infection results in myofibrillar breakdown in cardiac fibroblasts

To evaluate if *T. cruzi* induces cytoskeletal breakdown in cardiac fibroblasts, we stained normal and *T. cruzi* infected cardiac fibroblasts, treated or not with TGF-β, with phalloidin – FITC (4 μg/mL, Sigma Chemical Co.) to visualize actin microfilaments, together with regular FN immunofluorescence. Our results show that *T. cruzi* induces a disorganization of actin microfilaments (Fig S4 C-D), localized in the infected cell, when compared to normal cultures (FigS4 A-B) which present widespread actin fibril network. The breakage of microfilaments by the parasite is not affected by TGF-β treatment, where highly infected cells also present cytoskeleton breakdown (Fig S4 G-H). This phenomenon reflects in FN fibril organization, which is dependent of the cytoskeleton. CF cultures infected with *T. cruzi* shows FN networks distribution localized preferentially over uninfected areas of the culture (Fig S4 C-D), even after TGF-β treatment (Fig S4 G-H).

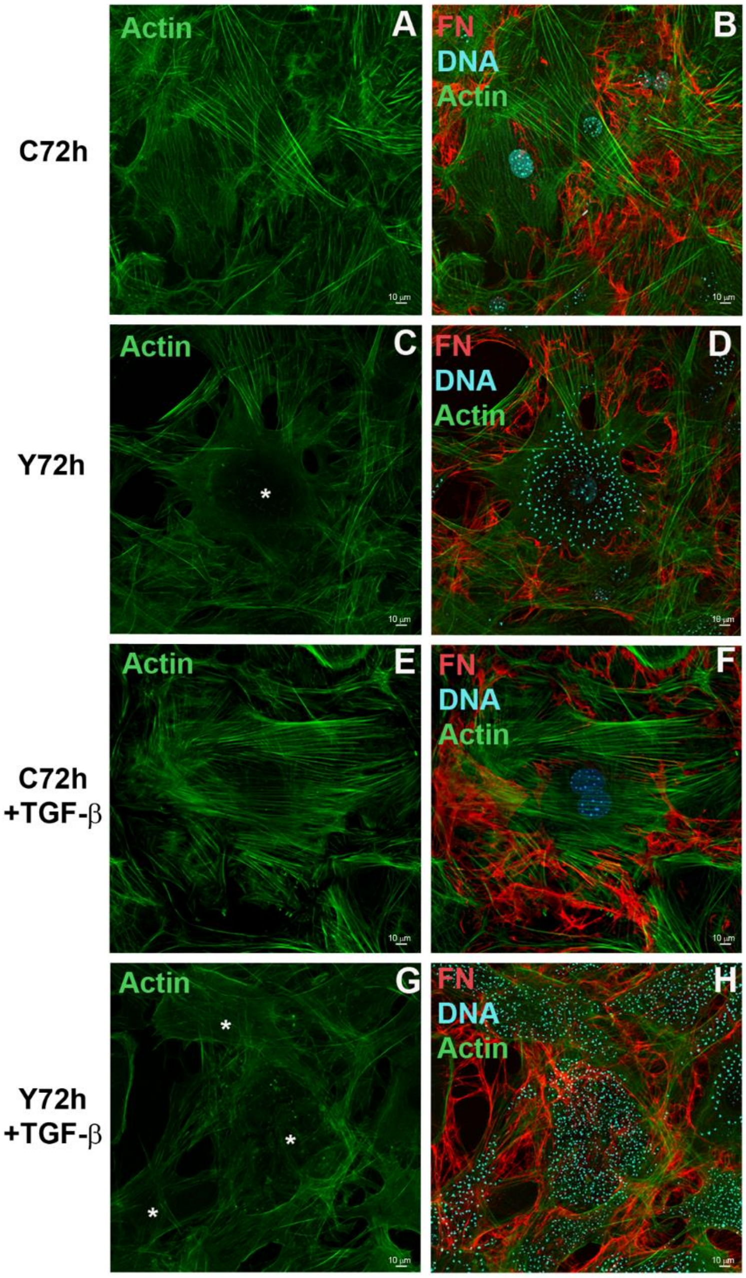


**Figure S4 – *Trypanosoma cruzi* disrupts actin cytoskeleton in cardiac fibroblasts.** Actin cytoskeleton was stained with phalloidin-FITC (green). DAPI (blue) was used to label the nucleus of the host cell and kinetoplast of intracellular parasites. Fibronectin immunostaining is represented in red. (A-B) Control uninfected cells; (C-D) *T. cruzi* infection disorganizes CF actin cytoskeleton; (E-F) CF treated with TGF-β showing an enhancement of FN (F); (G-H) TGF-β treated CF cultures still present actin myofibrillar breakdown and delocalization of FN network. *corresponding location of intracellular parasites. Bar = 10 μm.

**5. Negative controls for immunofluorescence assays**

Negative controls with omission of the primary antibody were made in parallel with all immunofluorescence assays and no unspecific staining was visualized with the secondary antibodies (Fig S5).

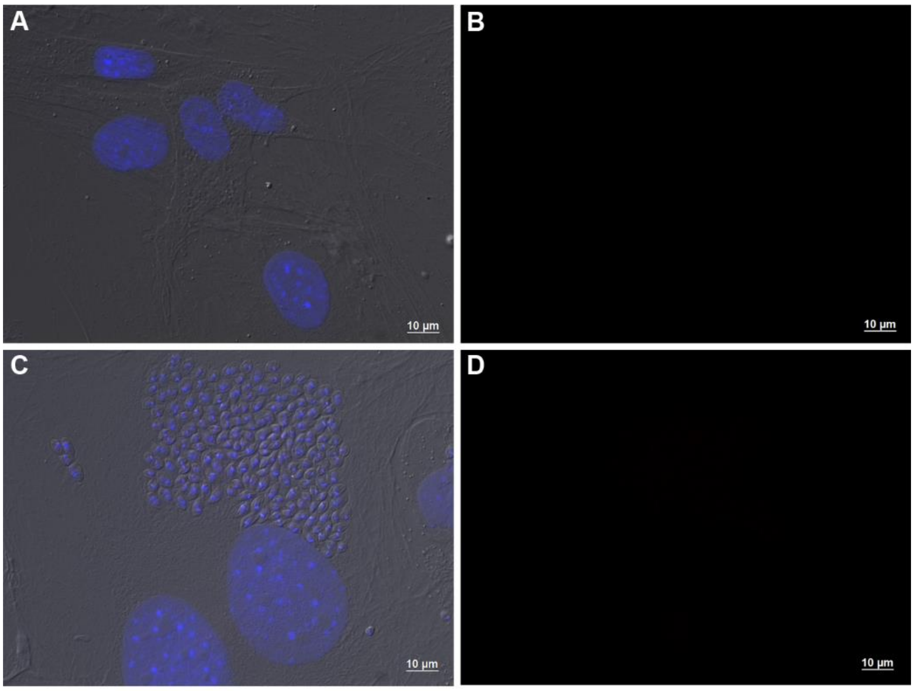


**Figure S5 – Negative controls for the immunofluorescence assays.** Negative controls for the immunofluorescence assays. (A-B) Uninfected cardiac fibroblasts; (B-C) Cardiac fibroblasts after 72h of *T. cruzi* infection. (A, C) DIC shows the general aspect of the cell in culture and DAPI (blue) stained the host cells nuclei and *T. cruzi* nuclei and kinetoplasts. Bar=10µm

## References

1. Calvet, C.M.; Oliveira, F.O.R.; Araújo-Jorge, T.C.; Pereira, M.C.S. Regulation of extracellular matrix expression and distribution in Trypanosoma cruzi-infected cardiomyocytes. *Int. J. Med. Microbiol.* **2009**, *299*, 301–12.

© 2019 by the authors. Submitted for possible open access publication under the terms and conditions of the Creative Commons Attribution (CC BY) license (http://creativecommons.org/licenses/by/4.0/).
